# Dispensaries

**Published:** 1834-01-01

**Authors:** 


### III. DISPENSARIES.

Shabbiness has had a temporary triumph; the Aldersgate street committee has found a set of medical tools, and consequently has gained the day, at the cost merely of ruining the institution to which they belong. It is but half a triumph, after all; for, in a cause like this, where the vanquished have the monopoly of good sense as well as good feeling, defeat, if it has not the advantages, has at least all the honours of victory. We look upon the secession of the late officers as the commencement of a happier era in the history of dispensaries, and trust that we shall soon see these institutions re-organized on a new basis. This basis should be public utility, to which all other considerations must yield. Why, for instance, should the sick poor be compelled to apply for relief exactly at a given hour? Surely a dispensary might be open, like a government office, from ten to four. The slaves of routine will answer, that the physicians and surgeons would be unwilling to stay six hours. True; but it would be easy to get active, zealous, paid officers, to remain three hours, and to come in rotation.

But there is another improvement which the state of society imperiously demands, and which is of still greater importance; we mean the establishment of self-supporting dispensaries. The mechanic, the small tradesman, the lean annuitant, could here find relief for their sufferings, without the degradation of receiving charity, or the painful anticipation of a "doctor's bill." From one to two guineas per annum, subscribed by each member of these medical clubs, would, we think, cover all the expenses of house-rent, medicine, &c. and leave a decent salary for each of the medical officers. The advantages are so obvious, and the thing is so feasible, that we have no doubt self-supporting dispensaries will soon be established in London. It is to be hoped, however, that the first founders may be persons of some weight and respectability, lest we should see the frail creations of their patronage vanishing like the Western Hospital; and again hear disappointed tradesmen exclaiming "Who is to pay us?"

[We have received the following article from a correspondent.—*Edit.*]

